# The Representation of Risk in Routine Medical Experience: What Actions for Contemporary Health Policy?

**DOI:** 10.1371/journal.pone.0048297

**Published:** 2012-11-01

**Authors:** Silvia Riva, Marco Monti, Paola Iannello, Alessandro Antonietti

**Affiliations:** 1 Catholic University of the Sacred Heart of Milan, Department of Psychology, Milan, Italy; 2 Institute of Communication and Health (ICH), Università della Svizzera Italiana, Lugano, Switzerland; 3 Max Planck Institute for Human Development of Berlin, Department for Adaptive Behaviour and Cognition, Berlin, Germany; The University of Hong Kong, Hong Kong

## Abstract

**Background:**

The comprehension of appropriate information about illnesses and treatments, can have beneficial effects on patients’ satisfaction and on important health outcomes. However, it is questionable whether people are able to understand risk properly.

**Aim:**

To describe patients’ representation of risk in common medical experiences by linking such a representation to the concept of trust. A further goal was to test whether the representation of risk in the medical domain is associated to the level of expertise. The third goal was to verify whether socio-demographic differences influence the representation of risk.

**Methods:**

Eighty voluntary participants from 6 health-centers in northern Italy were enrolled to conduct a semi-structured interview which included demographic questions, term-associations about risk representation, closed and open questions about attitudes and perception of risk in the medical context, as well as about medical expertise and trust.

**Results:**

The results showed that people do not have in mind a scientific definition of risk in medicine. Risk is seen as a synonym for surgery and disease and it is often confused with fear. However, general knowledge of medical matters helps people to have a better health management through risk identification and risk information, adoption of careful behaviors and tendency to have a critical view about safety and medical news. Finally, trust proved to be an important variable in risk representation and risk and trust were correlated positively.

**Conclusions:**

People must receive appropriate information about the risks and benefits of treatment, in a form that they can understand and apply to their own circumstances. Moreover, contemporary health policy should empower patients to adopt an active self-care attitude. Methodologies to enhance people’s decision-making outcomes based on better risk communication should be improved in order to enable low literacy population as well elderly people to better understand their treatment and associated risk.

## Introduction

Over the past two decades, there has been increasing recognition that people both want and need to be given accurate and understandable information about health [1]–[2]. In particular, people need to be told about the risks and benefits of their treatments in order to make informed decisions and effective choices [2]. This is a cornerstone of the philosophy of self-care and is a key element in current healthcare policy in the US and in many European countries [3].

In line with this, there is evidence that the comprehension of appropriate information about illnesses and treatments, including risk and side effects, can have beneficial effects on patients’ satisfaction and on important health outcomes [4]. Against this background, it is questionable whether people are able to be active and informed about risks and to take autonomous choices about their treatment. Indeed, people may not always be well equipped, either cognitively or emotionally, to understand, retain and use information effectively [5]. Often, provision of information on medicines does not always have beneficial effects and may even have unwanted or harmful effects on health [6]–[7]. In particular, informing people about the risks and benefits of possible treatments has become a major challenge for healthcare providers and general practitioners (GP). This is because the information is often complex, in that it can be ambiguous, incomplete, uncertain and unstable.

In clinical practice, a discrepancy has been demonstrated between patients’ individual perception of risk and GPs’ medical understanding of risk [8]–[9]. People exposed to the same objective risk can perceive it differently and give it dissimilar meaning in their everyday life. The GP’s duty is to attem pt to make the risk as understandable as possible and build a bridge between the patient’ subjective representation of risk and the objective risk [9].

In line with this, the role of trust in medical relationships represents a relevant aspect. Trust has been considered the lifeblood of the medical relationship [10]. It mediates positive outcomes, including adherence to treatment, improvement of knowledge, satisfaction and continuity of care [11].

### Risk and Trust

Various conceptualizations of trust have been offered over the years, with definitions covering notions as diverse as positive belief, personal trait, action, situational features and social structure [10]. In recent times, a construct that is increasingly being woven into the conceptualization of trust is risk. This risk-based approach to trust is gaining increasing acceptance among a number of theorists according to whom these two concepts appear related in several ways [12–13–14].

First, some researchers argue that only under conditions of risk is trust needed. In this sense, trust is defined as “an individual’s behavioural reliance” on another person under a condition of risk [12]. Second, researchers also suggest that trust can be viewed as an attribute of risk-taking behaviour [XPATH ERROR: unknown variable "start".]–. The willingness to take risks may be associated with a satisfactory level of trust between subjects. That is, a sense of trust encourages risk taking by trustors. In this frame, cognitive processes can also help to understand the link between trust and risk. In the field of cognitive psychology and decision making, several studies have documented how people’s perception of trustworthiness can directly influence attitudes and behaviour [15]–[16]. People often adopt the advice-taking heuristic to judge objects and make decisions in their environment; in other words, people engage themselves to collect relevant information, soliciting the opinions of worthy expert advisors in order to build their knowledge and to make choices [17]. Thus, when trust exists, advice-taking can represent a useful strategy to interpret medical risk and to appease doubts and fear.

Finally, some theorists point out that trust itself represents individuals’ perceptions of outcomes. Trust refers to the assessment of probability that the person will perform as expected [18]. For example, when a person thinks about her/his trust in a physician who is to perform a surgical procedure on her/him, trust is usually conceived in terms of the likely percentage of success. Confidence is measured by percentage because most people understand that there is nothing that is 100% certain in this world. Indeed, high levels of subjective trust only mean that the individual perceives the probability of having desirable performance from the other party as pretty high.

Once we accept subjective trust in terms of probabilities, the concept of risk becomes salient. The reason is that perceived risk is also the subjective estimation of probabilities [19]. In economics and psychology, risk has traditionally been meant as known probability, with uncertainty as unknown probability [8]. Although this difference is often blurred, perceived risk is generally regarded as calculative probabilities under conditions of uncertainty.

### Risk in the Medical Context: What Does It Mean?

From a scientific point of view, risk is amenable to a precise mathematical definition involving expectations, probabilities and utility functions [8]. The simplest definition that people should internalize as children is that a situation is risky when at least one possible event is connected to a loss of some resource or a negative consequence (e.g., a side effect for health). Perceiving and evaluating risk is based on two abilities:

assessing the likelihood of the hazard;
estimating the loss caused by the hazard.

Over the past years risk perception has become an increasingly relevant construct not only in economics but also in the medical context. Many studies on risk perception have investigated the effects of presenting risk information in different ways (for example, verbally, numerically or graphically [19]–[20], positively or negatively framed [21], relative or absolute forms of risk [22]–[23]) or in different orders (including the risk information at the beginning or at the end of the message [24]) or have looked at the perception of risky behavior and choices [25]–[26]. Despite the proliferation of these studies, the representation of risk in people’s everyday experience remains still unclear. Although in medicine we talk about risk specifically as a statistical concept [8]–[26], the layman could have a different understanding of what it concerns. Risk could mean different things to different people. Understanding the concept of risk means considering the relationship between a general definition and its implementation in concrete individual situations. It also means realizing the difference between risk as a statistical concept and risk as the content of the subjective meaning for the person.

The aim of this study is to investigate patients’ representation of risk in common medical experiences. More precisely, the goal was to link such a representation to the concept of trust, which is, according to the views reported above, critical for the subjective understanding of risk. A further goal was to test whether the representation of risk in the medical domain is associated to the level of expertise in medical issues. The third goal was to verify whether individual differences such as age, gender, education, job and health status influence the representation of risk.

## Methods

A semi-structured interview was designed to lead respondents to make explicit their perceptions and conceptions about risk in medical care and related topics.

### Participants

This research was conducted as part of a larger qualitative study into the meaning of “personal self-care” in the Autonomous Province of Trento (Northern Italy). Research Ethics Committee approval was granted for the study by the Italian ASL (Italian Primary Care Trust) of the Province of Trento.

For this study, 80 participants were sampled from the 6 main local ASL departments of Trento Province. Typically, such interview design involves conducting individual interviews with a small number of respondents to explore their perspectives on a particular idea, situation or person’s thoughts and eighty participants represent a large sample in this type of research [27].

The characteristics of the participants are summarized in Table 1. Except the youngest age group, the numbers of participants in the other age groups were similar. The sample was rather equilibrated according to gender and employment status. Most participants got a high school, or even higher, degree and were not affected by long-standing impairments or disabilities.

**Table 1:** Characteristics of participants.

Characteristics	N
*Mean Age, (range; SD)*	52.7 yrs. (24–79; 15.4)
*Age Groups*	
22–36 yrs.	12 (15%)
37–49 yrs.	25 (31%)
50–64 yrs.	23 (29%)
65+yrs.	20 (25%)
*Gender*	
Men	47 (58.8%)
Women	33 (41.2%)
*Education*	
Primary school	9 (11%)
Junior high school	10 (13%)
High school	46 (57%)
University	15 (19%)
*Employment Status*	
Employed	45 (56.3%)
Retired/	35 (43.7%)
Looking after family	
*Longstanding Impairment* *or Illness*	
Yes	10 (12.5%)
No	70 (87.5%)

We used a convenience sample. In pilot studies a convenience sample is usually used because it allows the researcher to obtain basic data and trends regarding his study without the complications of using a randomized sample. Furthermore, the structure of our sample can be considered sufficiently heterogeneous and representative according to the following conditions:

the epidemiological context of Trento region is similar to other Italian regions;
the health system procedures applied in Trento region are equal to other Italian regions;
the sample is heterogeneous by gender, age, level of education to the same extent as in other Italian regions;
the health operators of Trento region have the same qualifications as in other Italian regions.

We have no reason to suspect that possible biases affected the sampling procedure. The researchers contacted patients who came to the local ASL department in the same period of the year by asking them to volunteer in the study by taking part to an interview. About half of the patients were recruited in the morning and half in the afternoon, so that possible differences in job and family activities (which might lead people to prefer selectively a part of the day to come to the department because of the lack of duties) should be excluded. Patients were recruited in all the waiting rooms of the departments, so to exclude possible high rates of individuals showing a given pathology (associated to specific waiting rooms). Finally, we have used the same criteria to select cases and we have excluded self-referral cases.

### Interview Design

#### Section 1: Demographic characteristics

The interview started with a series of demographic questions, pertaining to the respondent’s age, gender, education, occupation and health (see the features of the sample and Table 1).

#### Section 2: Expertise

Participants were then asked about their personal knowledge of medical terms and their attitude and beliefs towards medical information:

- the respondent’s knowledge of medical terms;
- reactions to medical information (that is questions for understanding individual attitudes and differences toward medical information);
- personal beliefs about own expertise/naivety towards medical information;
- opinions about side-effects of their own treatments.

These issues were included in this section since, according to the more recent reviews in the field of health literacy and medical knowledge [28]–[29], the topic they make reference to are reliable indicators of knowledge and expertise in medical context.

#### Section 3: Trust

Two questions explore the role of trust in medical experiences:

*Do you have a long-lasting and trustful relationship with your GP? (Add any additional comment)*
*How much trust do you have in your GP? (Add any additional comment)*

These two questions were meant to check directly the patients’ levels of trust in their GPs.

#### Section 4: Risk

This section of the interview was constituted by two parts. First participants were invited to list three associations that usually came to mind when they thought about risk in a medical context. Answers were collected and lemmatized (e.g., drugs→drug), aggregated semantically (e.g., physicians, general practitioners, doctors→“doctor”; mistake, error→“mistake”) and were classified through the criterion of redundancy according to the quantitative content analysis. This technique, called the continued associations method, has been shown by Szalay and Deese [30] to be a sensitive indicator of the imagery and meaning associated with people’s mental representations for a wide variety of concepts.

Then participants were asked to indicate their perceptions, experience and opinions in response to risk understanding in the medical context. The questions concerned the following issues:

- perception of risk as linked with fear;
- perception of risk as linked with trust;
- the respondent’s personal experience of medical risk.

These issues were chosen since: (i) fear is often associated to the naïve conception of risk in different domains [31], and especially in medical context [32]; (ii), trust, as argued before, is a critical component of risk perception [12]–[XPATH ERROR: unknown variable "next".]; reporting personal experience sheds light on possible unexpected or implicit meanings associated to risk [33].

A map of the interview is described in Table 2.

**Table 2:** Map of the interview.

N°	Main Areas	Topic	Question	Type of questions
	SOCIO-DEMOGRAPHIC			
1		Age	How old are you?	Closed
2		Gender	(a) Female or (b) Male	Closed
3		Level of education	Which is your level of education? (a) Primary school, (b) Junior high school,(c) High school, (d) University	Closed
4		Job status	(a) Employed, (b) in retirement/Looking after family	Closed
5		Longstanding illness	Do you have any long-standing impairment, illness or disability?	Closed
	EXPERTISE			
6		Definition of medical terms	Response variability	Open
7			Skin Rash	Open
8			Amixocillina	Open
9		Self evaluation	Do you usually react to medical news concerning risks for health?	Closed
10			How do you consider yourself a naive patients in understanding medical information?	Closed
11			Do you consider yourself an expert patient in dealing with medical problems?	Closed
12			In your opinion, was your treatment safe and not risky according to the information that you were aware of?	Closed
	TRUST			
13		Long-term relationship	Do you have a long-lasting and trustful relationship with your GP?*(Add any additional comment)*	Closed
14		Trust in GP	How much trust do you have in your GP? *(Add any additional comment)*	Closed
	RISK			
15		Representation of risk	Word associations	Word association
16		Risk and fear	In your opinion are risk and fear linked together? (*Add any* *additional comment)*	Closed
17		Risk and trust	In your opinion, do you perceive risk and trust as linked together? If so,in which sense?	Close+Open
18		Risk and experience	Did you face any experience about risk (eg. negative experience, risk of a treatment followed, drug assumed) to tell us? If so, what did you GPdo with you?	Open
19			Which information could help you in better understanding risks associatedwith a treatment?	Open
				
20			Which aspects could improve the quality of communication with yourphysician in terms of risk comprehension?	Open

Closed questions asking to rate the agreement toward a given sentence or to express an evaluation were based on an 11-point scale from “not at all” to “completely”, as follows (Table 3):

Answers to closed question could be enhanced by personal comments about individual experiences or opinions.

**Table 3:** Likert scale.

Not at all		Mostly not		Somewhat			Mostly yes		Completely	
1	2	3	4	5	6	7	8	9	10	11

### Administration of the Interview

The intention was to interview respondents in a naturalistic and familiar environment. Some medical rooms of the primary care service were used for conducting interviews. Interviews were conducted by 3 social scientists trained in qualitative research (2 of whom are authors of this article, S.R and M.M.). Participants signed an informed consent to declare their participation in this research.

To facilitate the data collection and the subsequent analysis, the interviews were audio-registered and transcribed using Unipark [34], a qualitative interview software for empirical research. This allowed us to track results and check for possible interactions and misunderstandings and to organize better the file of the answers using different formats.

## Results

### Expertise

Questions included in the second section of the interview tested general knowledge about health, disease, side-effects and treatment. When asked to define some medical terms, the sample’s answers differed greatly. Out of 80 participants, 38 (47.5%) gave two correct answers but only 12 participants gave three perfect definitions. More than half of the sample (52.5%) gave only one or no complete answers.

In order to define better these intra-group differences and to clarify the different levels of knowledge, a K-mean clustering analysis was performed. This procedure attempts to identify relatively homogeneous groups of cases based on selected characteristics using an iterative algorithm. This technique is an exploratory data analysis tool which aims at sorting different objects into groups in such a way that the degree of association between two objects is maximal if they belong to the same group and minimal otherwise.

**Figure 1:**
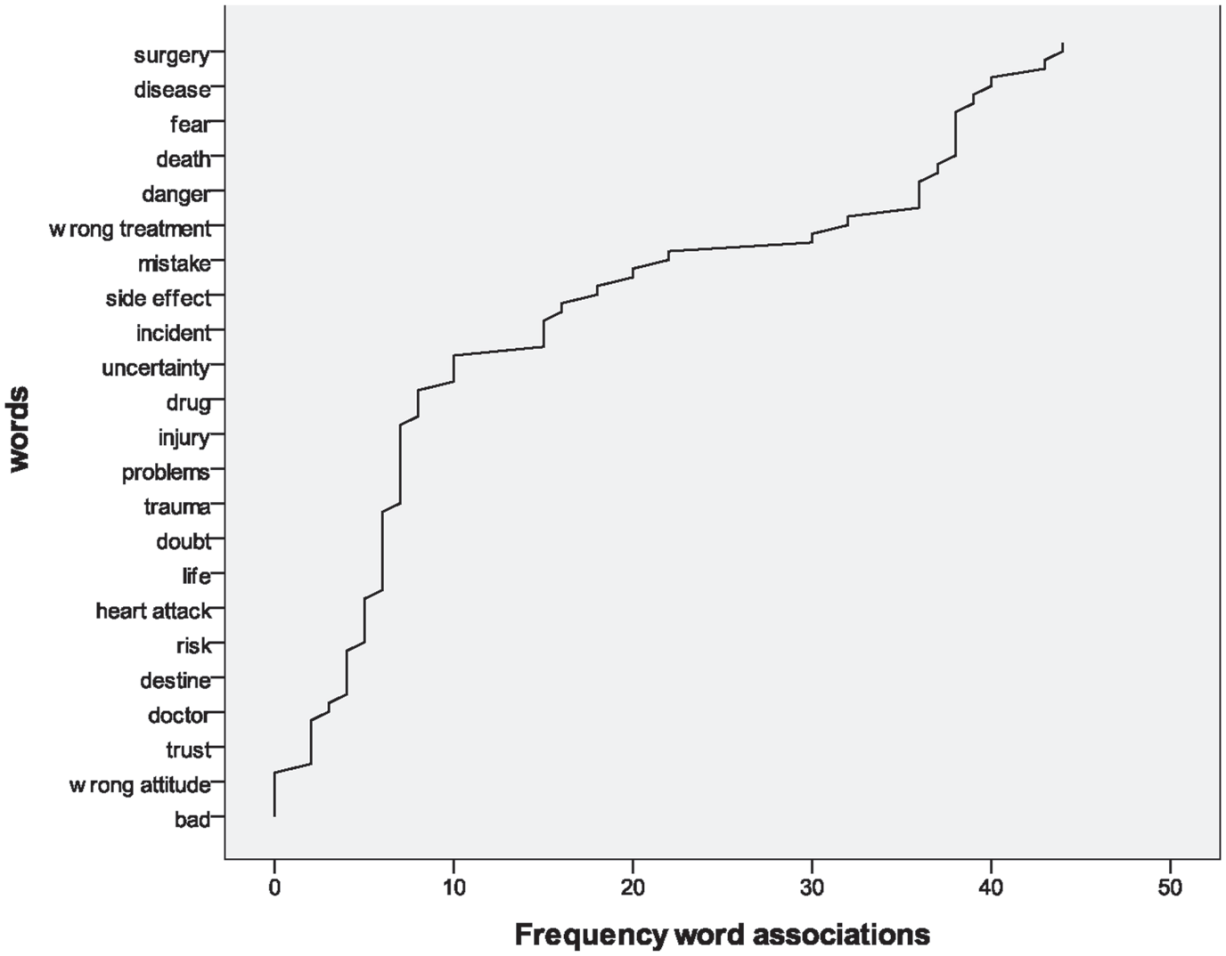
Diagnostic Box-Plot.

The cluster analysis led us to identify 2 homogeneous clusters, as shown by the diagnostic box-plot (Figure 1).

Cluster 1 (N = 48) included people with a good level of expertise in medical matters. Indeed, these participants did not consider themselves as naïve patients, usually reacting to medical news and information concerning risks for health and appearing more critical about the safety of their past treatments. All the 38 subjects who defined medical terms correctly were inserted in Cluster 1. On the contrary, Cluster 2 (N = 32) included people with a lower level of expertise. Indeed, these participants considered themselves more naïve than the other group and had greater difficulties in dealing with medical information, usually reacting less to medical news concerning risk for health and consequently appearing more confident about the safety of their treatment (Table 4 and Table 5).

**Table 4:** Cluster analysis.

Key features	Cluster 1 means	Cluster 2 means
Do you usually react to medical news concerning risks for health?	9	3
Do you consider yourself a naive patients in understanding medical information?	3	8
Do you consider yourself an expert patient in dealing with medical problems?	8	2
In your opinion, was your treatment safe and not risky according to the information that you were aware of?	5	9

**Table 5:** ANOVA from cluster analysis.

ANOVA						
	Cluster		Error			
	Squares Mean	df	Squares Mean	df	F	Sig.
Do you usually react to medical news concerning risks for health?	1.26	1.00	1.36	78.00	9.25	.003
Do you consider yourself a naive patients in understanding medical information?	54.40	1.00	1.36	78.00	399.55	.000
Do you consider yourself an expert patient in dealing with medical problems?	15.40	1.00	0.74	78.00	20.80	.000
In your opinion, was your treatment safe and not risky according to the information that you were aware of?	21.73	1.00	2.54	78.00	85.35	.000

Considering socio-demographic characteristics, no significant differences emerged between the two clusters in relation to age, gender, occupation and longstanding impairments. Only the level of education had an influence: the majority of people with low education were included in Cluster 2.

### Word Associations with Risk

**Figure 2:**
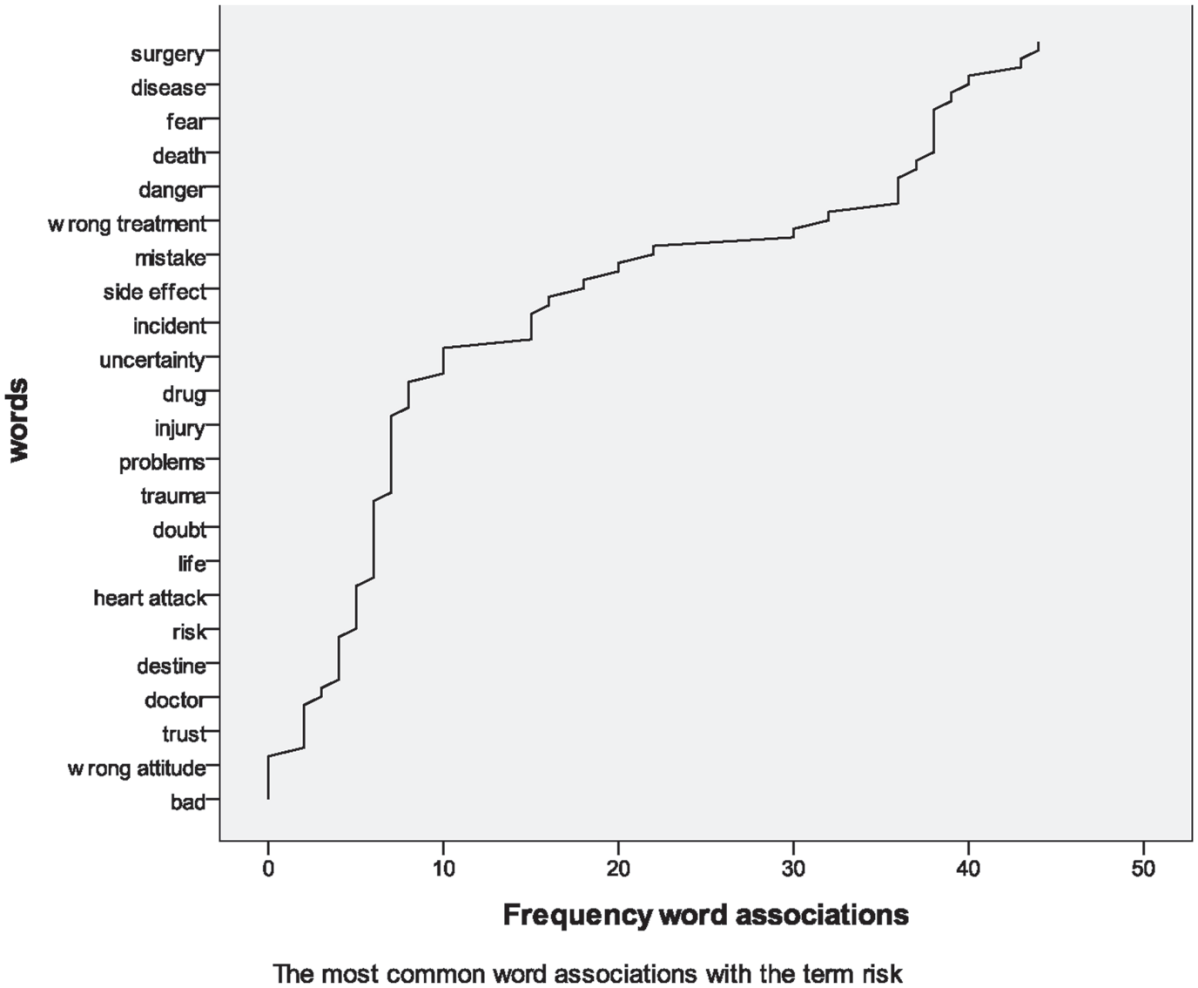
Word associations with the term “risk”.

More than 250 associations were produced in response to the stimulus concept “risk”. The major types of associations are described in Figure 2 in order of their frequency. Word associations revealed negative connotations of the term “risk”. In particular, risk was perceived as associated with the term “surgery” and “disease”. Risk was also misunderstood as an emotion of fear. Some participants associated negative results such as wrong treatment or side-effects and other participants underlined the negative effects of risk like danger, uncertainty and incident. Some participants associated the term “risk” with a concrete disease (e.g., heart attack or injury). None of the most frequently mentioned terms corresponded to a statistical or scientific definition of risk.

Although the definition of risk is not unique, according to classical definitions it involves expectations, probabilities, potential loss. Risk, generally, is something that can be framed, calculated or measured and it can be expected. However, results showed that the representation of risk for the respondents was far from a statistical concept. Risk is something that cannot be measured or calculated and it is never expected.

No link between the clusters based on medical expertise and the frequencies of the different associations emerged.

### Attitudes to Risk

#### Risk and fear

As outlined by the results on word associations, the rate of answers confirmed that risk strongly brings to mind fear and disease: 75% of the participants considered these two terms as linked together; a risky situation inevitably determines fear. Moreover, fear causes difficulties in conceptualizing risk in medical choice concretely, as reported by the 34% of the participants.

#### Risk and trust

**Figure 3:**
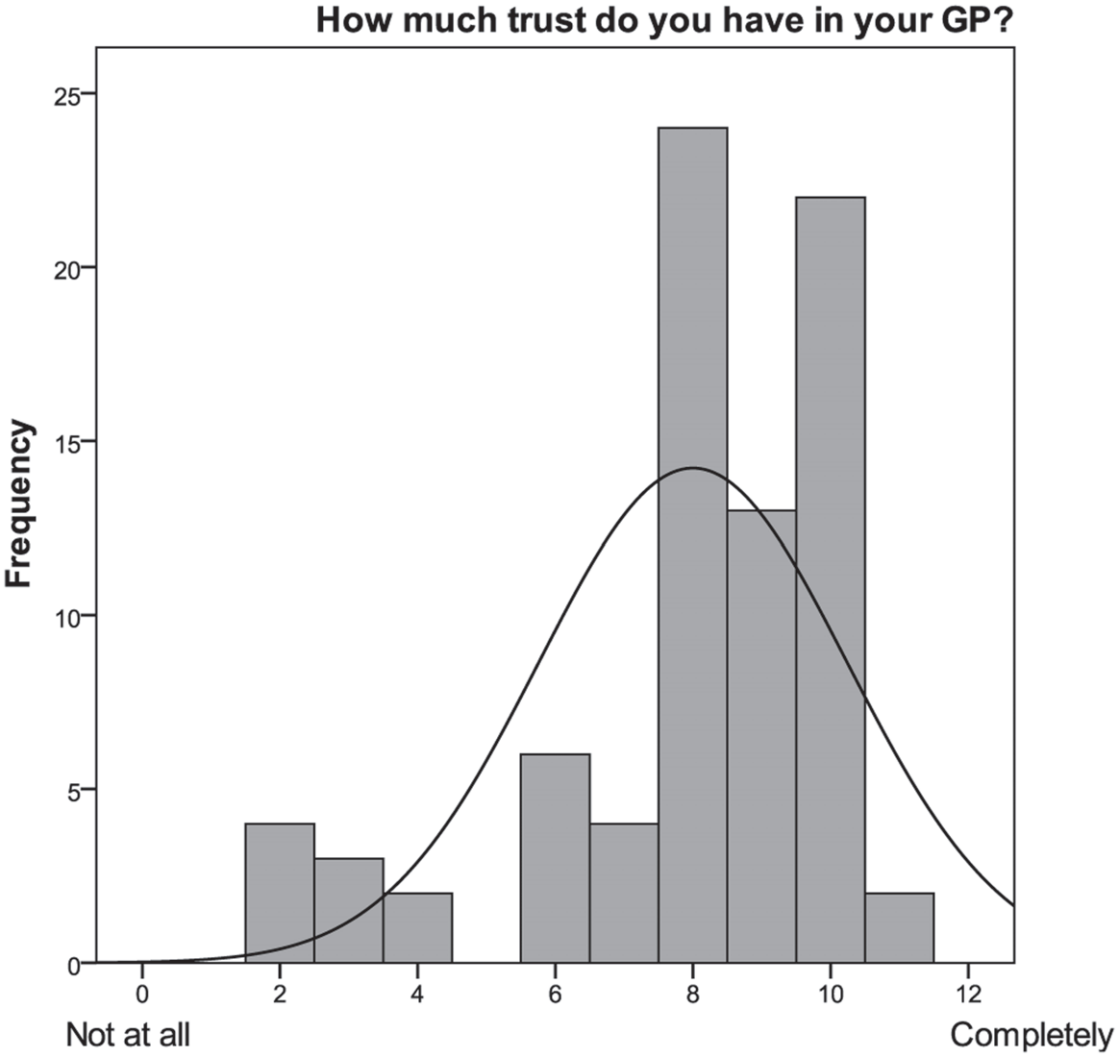
Frequency histogram of the item “How much trust do you have in your GP”?

**Figure 4:**
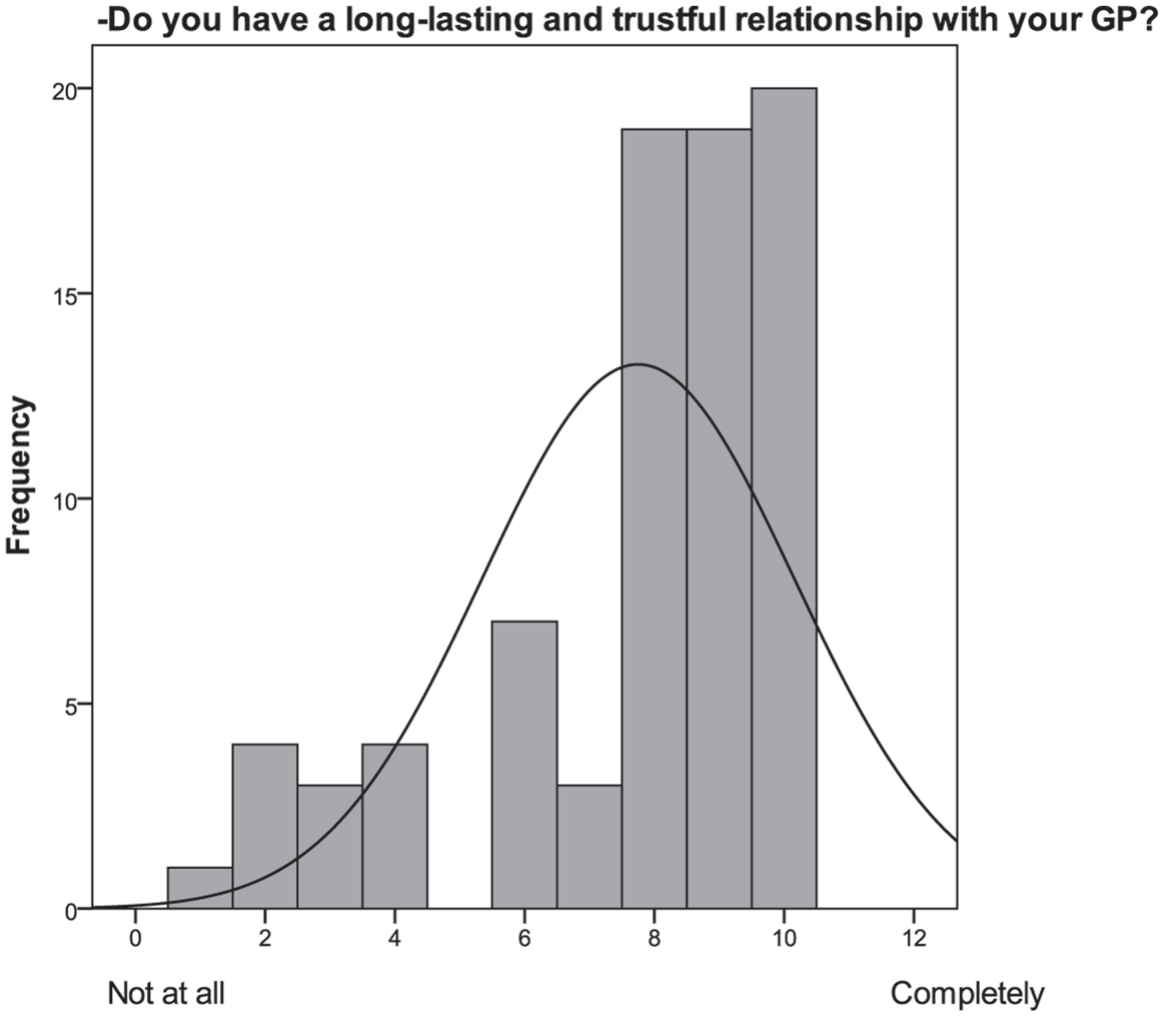
Frequency histogram of the item “Do you have a long-lasting and trustful relationship with your GP”?

In the third section of the interview participants were asked to indicate whether they trusted their GP and believed they had a long-lasting and trustful relationship with him/her. The great majority of participants (76%) showed a high level of trust and perceived a long-lasting and trustful relationship with their GP (Figure 3 and Figure 4). Participants were likely to report high trustful relationship with ratings of 8 or 9 on the 11-step scale, where 8 and 9 represented very high degree of trustful interaction. Considering the cluster analysis, no differences emerged in trust, which had constantly high rates in both groups.

Participants rated the correlation between trust and risk as very high (8 or 9 on the 11-step scale) for nearly half of the sample (N = 36; 45%). Trust and risk were found to be positively correlated (r = .30, p<.05). Further comments enhanced the description of the bound between trust and risk. All the comments were transcript and read multiple times. From this initial review of the transcript, the researcher (S.R. M.M.) begun to see themes emerging from the data and categorized the answers [35]. Collecting additional comments, it emerged that trust and risk were strictly linked for the 48% of the sample because trust (in the doctor) deadens fear (of a disease, of a course of drugs, for example) and doubts. According to our results, trust is especially reassuring for patients who know that they are at high risk of developing a disease (e.g., participants at risk of developing a cardiovascular disease) or people who do not follow preventive or health behaviors (e.g., diet or exercise).

#### Risk and personal experience

Participants were asked about their personal experiences of risk and risk comprehension through three open questions, as detailed in Table 1.

As above, S.R. and M.M. independently read all of the transcripts and developed an initial list of codes. The independent coding was subsequently jointly reviewed by the analysts. Transcripts were then reread to confirm the list of codes and create subheadings [35]. The constant comparative method approach was employed to ensure that the analysts defined and applied the codes in a consistent manner across all transcripts. Initial open codes were generated from the text. Disconfirming cases were sought. Codes were grouped into a set of organizing themes, then storylines and concept maps [35] were used to identify relationships between subthemes. Using two coders ensured that a broader range of codes was identified. T-LAB [36] was used to identify and sort the relevant text across the transcripts for each code.

#### Main themes

Four elements were repeatedly described by respondents as being essential to enrich patient’s comprehension and knowledge of treatment and risks associated: (1) Clearness (2) Sharing information, (3) Time, (4) Continuity. As illustrated in the following paragraphs, these domains overlap, but each emphasizes distinct goals that must be fulfilled to make patients more aware of their health and treatment.

*-Clearness of treatment information*

Participants repeatedly emphasized that being informed in a clear and simple way is absolutely essential to be able to understand treatment and possible risks:

I perceive his honesty and sense of realism through his calm and clearness (Interview 35).

One time I was uncertain about the use of cortisone but my GP reassured me, he explained the risks and the benefits of using this drug to me and, at the end, I had a very good outcome and I solved my problem (Interview 10).

Several participants pointed out that they had considerable difficulty dealing with the amount and complexity of the information they received:

When my wife was diagnosed with LES, the GP explained the disease to me in simple words because this disease is really complicated and has different effects. When my wife feels bad, I generally phone my doctor first. He is really very competent (Interview 24).

-*Sharing information*

Beyond providing and clarifying complex information, participants in this study felt that physicians should facilitate patient knowledge by sharing information with patients:

Generally, my GP takes his time to explain the problems to me, he phones me at home if necessary and he encourages discussion and sharing of the problem (Interview 14).

Other respondents emphasized the patient–physician relationship as being an important partnership in which to evaluate choices and make decisions:

Well, I’m very confident in the doctor…. One time, he identified my appendicitis inflammation, well. He perfectly explained to me what to do before a possible operation (e.g., type of diet, how to recognize the typical pain of appendicitis, where to go) (Interview 8).

My doctor asked me what I might prefer when the menopause occurred and she explained HRT - Hormone replacement therapy to me (Interview 66).

*-Time*

The lack of time spent with patients during medical encounters was frequently cited as a barrier to providing effective healthcare. In this study, at the end of the interview, when specifically prompted to describe additional barriers to enabling active patient participation in understanding and managing their treatment and risks, time was one of the most important factors discussed by participants. Limited time was seen both as a barrier to becoming informed and as a barrier to evaluating risks, problems and fears.

A lot of them don’t explain things–they don’t have the time or they don’t take the time. (Interview 12).

It just doesn’t feel like there’s ever room in the system anymore for real dialogue. In other words, that’s what gets in the way. (Interview 3).

Time…Time sadly. (Interview 5).

I do not have the chance to look his face when I go to him….the visit is too short. (Interview 1).

Participants also felt that lack of time limits the extent to which physicians can help their patients to process information.

They (patients) bring things in from the Internet, and then time is taken up wading through a lot of stuff, which may not even be of importance. So…when there is a little bit of time it is confused by all of the outside information that patients have. (Interview 11).

*-Continuity.*

Consulting a GP repeatedly provided the opportunity to amass knowledge and monitor the therapy (or the treatment) during the ongoing process. Two features seemed especially important to patients as elements of a doctor’s trustworthy behavior over time: the extent to which GPs seemed competent and the extent to which they appeared to act in the patients’ interests.

My doctor was very active when I had this viral infection….he gave me not only good treatment but he also phoned me at home during the weekend because he wanted to know how I felt…he maintains a good relationship with me (Interview 37).

Participants feel themselves safe and far from risks when their GP seems intrinsically motivated to care for patients, is willing to invest effort in the patient, shows personal knowledge, is particularly caring, invests time in identifying and resolving their problems or makes an additional effort to help them.

Generally, my GP takes his time to explain the problems to me, he phones me at home if necessary. (Interview 70).

When we discovered that my son had hemophilia, our doctor was really close to us; he gave us a lot of information about this disease, the contact number of the hemophilia center. And anytime my son has to go to the emergency department for a hemorrhage…our doctor is always informed and he always calls us at home. He is really supportive. (Interview 80).

## Discussion and Conclusions

Increased emphasis on self-care and on disease prevention has shifted responsibility to patients, who now more than ever need to understand treatment information so as to actively participate in making decisions about their health1. Understanding treatment implies taking into account a quantity of information about the disease and the possible associated risks. In this study, we addressed the problem of how people represent the concept of risks in medical experience.

The results showed that people do not have in mind a scientific definition of risk; risk is seen as a synonym for surgery and disease and it is often confused with fear. For some participants, risk was conceptualized as wrong treatments or side-effects; for other participants risk was represented by danger, uncertainty and accidents. Risk was also associated with a concrete disease (e.g., heart attack or injury). None of the terms most frequently mentioned by respondents corresponded to a statistical or scientific definition of risk. Contrary to the definition in classical literature, risk is not conceived as something involving expectations, probabilities or utility [14]–[15]. Risk is not framed, calculated or measured. People seem far from a classical-statistical definition of risk. This result is comparable with previous evidence reported by several authors who pointed out how people can have complex and ambivalent views of associated risk and that they may not be well equipped, either cognitively or emotionally, to understand risk effectively [10]–[12].

Despite this representation, knowledge is a positive support for risk comprehension. A general knowledge of medical matters helps people to have better health management through risk identification and risk information, adoption of careful behaviors and a tendency to take a critical view about safety and medical news. Conversely, as shown by cluster analysis, low-literacy people were more uncertain about risk information and treatment safety. Unlike other studies of risk perception [37], no gender or age-group differences were found about risk information and medical knowledge, while the level of schooling was decisive. This is in line with other studies where scarce knowledge and low literacy clearly affect risk perception [38]–[39]. These patients seem to have less precise mental representations of risk [38]–[40].

This research also outlined the role of trust in GPs in risk representation. Participants showed a high level of trust and half of them considered trust and risk to be linked together, showing a significant positive correlation. The role of trust, in risk analysis, has not been addressed frequently in medicine, forcing us to speculate on possible processes. One explanation is that patients who have trust in their GP may have more confidence that the physician will detect, diagnose and treat a disease successfully. Indeed, trust was found to be especially reassuring for patients who believe that they are at high risk of developing a disease or people who follow prevention or maintain healthy behaviors. This reassurance might act as a type of social support that buffers (i.e., moderates) the detrimental effects of a stressor (i.e., perceived risk or negative medical news) on their health [16]. Conversely, low levels of trust among patients who have high risk perceptions may be especially stressful and therefore damaging to their health [9]. This result, however, supports the theories that conceive risk and trust as being strictly related.

In particular, as Das suggested, trust represents “an individual’s behavioral reliance” on another person under a condition of risk, and he argued that under certain conditions of risk (e.g., risk from a course of drugs), trust is needed [13]. In accordance with cognitive theories, people seem to adopt the advice-taking heuristic to judge their experiences, problems or possible risks, soliciting the opinions of worthy expert advisors [15]–[16]. The simple social heuristic “trust your doctor” becomes ecologically rational in environments where physicians understand health data [34] and when they are able to guarantee patients’ safety.

Finally, extensive analysis was done to deepen participants’ medical experiences of risk through some open questions. Four elements were found to be essential to enrich patient comprehension and knowledge of treatment and risks associated: (1) Clearness of treatment (2) Sharing information, (3) Time, (4) Continuity. Participants repeatedly emphasized the importance of being adequately informed about drugs and treatment. They also stressed that merely obtaining clear information was necessary, but not sufficient, to become a careful patient. To achieve the latter, resources are needed to help patients to understand medical information and the possible associated risks. In particular, open communication founded on sharing information and availability of time was found to be a core aspect for our participants.

This is an initial study and the present findings require further investigation. In fact, despite the desire to examine the views of patients in depth, it must be acknowledged that the participants included in the study represent only a small group of patients from a single Italian region. They might not be representative of patients with specific chronic illnesses or who usually consult only specialists. Furthermore, it is important to compare our results investigating the view of patients’ in longitudinal studies in order to evaluate how risk is conceived over time in relation to possible changes and critical events.

In spite of these limitations, it is hoped that the framework from this study is helpful to health providers, such as doctors, but also researchers who work in the field of risk communication and health policy, because it analyses some aspects that are not yet well described by current literature.

In the contemporary society of self-management, if medicines and treatment are to be taken safely and effectively, people must be given appropriate information about the risks and benefits of them, in a form that they can understand and apply to their own circumstances. Moreover, if the comprehension of risk is unclear, contemporary health policy should empower people to an active attitude of self-care: they should be encouraged to identify relevant information, such as details about what the treatment is for, how it should be taken, important contraindications and other warnings, and possible adverse effects.

The use of heuristics can help patients in this activity. Heuristics are simple decision strategies that ignore part of the available information, basing decisions on only a few relevant predictors. As recently confirmed by other studies [41]–[42], heuristics have various general features like accuracy, transparency, and wide accessibility, as well as low costs and little time required to employ them, all of which render them especially suitable tools to improve applied medical decision making. Embracing this emphasis on simple decision strategies and their fit to the environment, people could be empowered to recognize and use such heuristics properly.

Last but not least, in a contemporary health policy perspective, it is not only necessary to consider what information to give to people, how to present it, and in what order, but one also needs to take account of how it is processed. In this perspective, in the context of cognitive psychology, there is great potential for what is called “the architecture of choice” [43]; that is, decisions are influenced by how the choices are presented. One possible way to improve people’s decision outcomes is to design information environments that support transparent communication. For example, providing a clear list of the pros and cons of a decision can guide patients in formulating their own such list. Information can be directly improved in the main places where people usually purchase drugs, such as pharmacies but also supermarkets or where the GP sees their patients.

Furthermore, recent studies in the field of cognitive psychology and health prevention have shown the potential power of some methodologies like visual displays and numerical graphs employed to communicate risk reductions. Presenting numerical information regarding risk in imaginable and identifiable formats could encourage and enable people to think about it in an active and deliberate way [1]–[17].

Adhering to these recommendations clearly will not guarantee the safe and effective use of all medicines. However, it should prevent many of the common misunderstandings that currently occur, and could help patients to understand the key information that they need to make appropriately informed choices and to use their drugs in the intended manner.
